# Bmi-1 promotes the proliferation, migration and invasion, and inhibits cell apoptosis of human retinoblastoma cells via RKIP

**DOI:** 10.1038/s41598-024-65011-6

**Published:** 2024-06-24

**Authors:** Qian Li, Te Fu, Ning Wei, Qiaoling Wang, Xin Zhang

**Affiliations:** Department of Ophthalmology, The Second People’s Hospital of Jinan, No. 148, Jingyi Road, Jinan, 250000 Shandong China

**Keywords:** Bmi-1, RKIP, Retinoblastoma, Tumorigenesis, Predictive markers, Eye cancer, Cancer, Oncogenesis, Medical research, Molecular medicine, Oncology

## Abstract

Retinoblastoma is one of the most common ocular malignancies in children. Bmi-1, a member of the Polycomb group family of transcriptional repressors, is expressed in a variety of tumors. The purpose of our study was to explore the role of Bmi-1 in retinoblastoma. RT-qPCR and western blot were used for calculating the mRNA and protein levels of Bmi-1 and RKIP. MTT, Wound healing and Transwell assays were performed to measure the proliferation, migration and invasion in retinoblastoma cells. Cell apoptosis was detected by flow cytometry. The volume and mass of transplanted tumors were detected in nude mice. Bmi-1 was over expressed, and RKIP was low expressed in retinoblastoma cells. Bmi-1 promoted cell proliferation, migration and invasion and suppressed cell apoptosis of Y79 and SO-RB50 cells. Downregulation of Bmi-1 and overexpression of RKIP inhibited cell proliferation, migration and invasion, and increased cell apoptosis. The functions of Bmi-1 knockdown on retinoblastoma cells were blocked by RKIP knockdown, but promoted by RKIP. Down-regulated Bmi-1 inhibited xenograft tumor growth, and RKIP exacerbated this inhibitory effect. Bmi-1 served as a potential therapeutic target for improving the efficacy of clinical treatment in retinoblastoma. All the findings revealed the functions of Bmi-1/RKIP axis in retinoblastoma tumorigenesis.

## Introduction

Retinoblastoma, one of the most common ocular malignancies in children, occurs mostly in the nuclear layer of the retina and has a certain family tendency^1^. Retinoblastoma is more common in children under 5 years old, and is prone to intracranial and distant metastasis, which seriously threatens the life and health of children^2,3^. The existing treatments for retinoblastoma include chemotherapy, laser photocoagulation, cryotherapy, radiotherapy, and enucleation^4^. The preferred treatment option is systemic chemotherapy combined with local consolidation therapy, such as photocoagulation and cryotherapy^5^. However, this treatment method has poor efficacy in patients with late retinoblastoma, and chemotherapy is accompanied by serious side effects and with the problem of chemotherapy resistance^6^. In recent years, new discoveries in tumor molecular biology have emerged endlessly, making gene therapy a treatment method with broad application prospects. Exploring the molecular mechanism of retinoblastoma, finding biological markers for early diagnosis and new therapeutic targets are of great significance for improving the diagnosis and treatment level of retinoblastoma and improving the prognosis of children^7,8^.

B lymphoma Mo-MLV insertion region 1 (Bmi-1), a member of the Polycomb group family of transcriptional repressors, regulates cell cycle progression and prevents cell senescence by inhibiting the p16/Rb and p19/p53 pathways^9,10^. Bmi-1 is expressed in various tumors and is closely related to tumor occurrence, development and prognosis^11,12^. Studies have shown that the occurrence and development of retinoblastoma are related to various molecular mechanisms, such as gene mutations and epigenetic modifications, and Bmi-1 may play an extremely important role in this process^13^. Bmi-1 is related to tumor size, clinical stage and prognosis of gastric cancer^14^. Additionally, we proved that Bmi-1 is negatively correlated with Raf kinase inhibitor protein, chemotherapeutic-induced apoptosis regulators and clinically relevant cancer metastasis suppressor genes^15^. Previous reports have shown that Bmi-1 maintains the self-renewal of adult progenitor cells or stem cells in different adult organs or tissues^16,17^. In this study, RNA interference technology was used to suppress the expression of Bmi-1, and to observe the inhibitory effect of Bmi-1 siRNA on the expression of Bmi-1 gene in human retinoblastoma cells and its effect on cell proliferation, migration, invasion and apoptosis.

## Materials and methods

### Cell culture

RMPI-1640 and MCDB-131 medium containing 10% fetal bovine serum were used to culture human retinoblastoma cell line Y79, SO-RB50 and Weri-RB1, and human normal retinal vascular endothelial cell line ACBRI-181 (ATCC, USA). The culture solution is cultured in a 37 °C, 5% CO_2_ constant temperature incubator.

### Cell transfection

The transfection operation was carried out using Lipofectamine 3000 according to the manufacturer’s instructions. siRNA-Bmi-1 (siBmi-1-1: 5′-CCAGAUUGAUGUCAUGUAUTT-3′; siBmi-1-2: 5′-ATATGAAGAGAAGAAGGGATT-3′) and siRNA-RKIP (siRKIP-1: 5′-TGGTCAACATGAAGGGTAA-3′; siRKIP-2: 5′-CAGCCACTTTCGCTATTCTTGTGTT-3′) were synthesized by GenePharma Company (CHN), Bmi-1 overexpression (pcDNA3.1-Bmi-1) and RKIP overexpression plasmid (pcDNA3.1- RKIP) were purchased from SinoBiological Company (CHN).

### Real-time quantitative polymerase chain reaction (RT-qPCR)

After 48 h transfection, total RNAs were collected and extracted by using the TRIzol reagent (Thermo Fisher, USA). The reverse transcription was performed to synthesize the first cDNA chain using cDNA first-strand synthesis kit (Vazyme, CHN). Finally, 2 μL cDNAs were taken to perform the qPCR using the SYBR Green master kit (Thermo Fisher, USA). The reaction conditions were pre-denaturation at 95 °C for 30 s, denaturation at 95 °C for 5 s, annealing and extension at 60 °C for 30 s, and amplification for 40 cycles. 2^−∆∆Ct^ method is used to calculate the relative mRNA level of Bmi-1. β-Actin was used as an internal reference. The primer sequences are in Table 1.

**Table 1:** Primer sequences in RT-qPCR.

Gene		Primers 5′–3′
Bmi-1	Forward	5′-GTGTGTGCTTTGTGGAGGGTAC-3′
	Reverse	5′-GTGGTCTGGTCTTGTGAACTTGG-3′
RKIP	Forward	5′-AGCAGTGGCACAGTCCTC-3′
	Reverse	5′-TGGTCTCCAGATCGGTTG-3′
β-Actin	Forward	5′-GAGACCAGGTTGTCTCCTG-3′
	Reverse	5′-GGTGGAATTGTGAGGGAGA-3′

### Western blot

After 48 h transfection, RIPA buffer (Beyotime, CHN) was applied to collect the protein of samples. SDS-PAGE was utilized to separate equal amounts of samples, then transferred onto polyvinylidene difluoride (PVDF) membranes. After blocking with 5% non-fat milk in TBS/0.5% Tween-20 (TBS-T) for 1 h at room temperature, the membranes were incubated with primary antibodies overnight at 4 °C. The primary antibodies were Bmi-1 Rabbit pAb (1:5000, ABclonal, CHN), RKIP Rabbit pAb (1:1000, ABclonal, CHN) and β-actin and a Rabbit mAb (1:50,000, ABclonal, CHN). Afterwards, horseradish peroxidase (HRP)-conjugated secondary antibodies (1:5000, ABclonal, CHN) were used for incubation of the membranes. ECL Enhanced Plus chemiluminescent substrate (ABclonal, CHN) was applied to detect the HRP and the blot was visualized in an UVP Bioimaging system. ImageJ software was used to quantify the protein levels.

### Cell proliferation

The MTT method was used for detection, and the cells in the logarithmic growth phase were used to perform the experiment. A single cell suspension with a cell number of 1 × 10^5^/mL was prepared and seeded in a 96-well plate at 200 μL/well. After 24, 48 and 72 h, cell viability was measured by MTT. 4 h before the end of the experiment, 20 μL of 5 mg/mL MTT was added to each well, and the incubation continued for 4 h. After the experiment, the culture solution was centrifuged and discarded. The microplate reader detects the absorbance of each well at a wavelength of 490 nm.

### Transwell assay

Transwell assay was applied to test cell invasion ability at 24 h after transfection. In brief, non-serum RMPI-1640 Medium was used to prepare single cell suspensions at a cell concentration of 3 × 10^4^ cells/mL. Before invasion assay, the upper chamber was covered with Matrigel (Millipore, USA). The upper chamber was added 0.1 mL cell suspension, while the lower chamber was filled with medium containing 20% FBS. After cultivated for 12 h, the cells under the lower surface of upper chamber was stained with 0.5% crystal violet (Beyotime, CHN) for 15 min at room temperature. Afterwards, an optical microscope was employed to observe and count the invasive cells.

### Flow cytometry (FCM)

After 48 h transfection, the cells were trypsinised. After digestion, the cell pellet was collected by centrifugation at 1200 g for 5 min. Cells were resuspended and washed twice with pre-cooled phosphate-buffered saline (PBS). Subsequently, depending on the volume of the cell pellet, appropriate amounts of Annexin V-FITC and propidium iodide (PI) (Beyotime, CHN) were added and incubated for 3 and 10 min respectively. Finally, a flow cytometer (BD Biosciences, USA) was utilized to detect the cells apoptosis.

### Xenograft assay

Four-week-old BALB/c nude mice were used for tumor transplantation. Mice were kept under sterile conditions with 12 h of light followed by 12 cycles of darkness. siRNA-Bmi-1 or RKIP were loaded into lentiviral vectors (), which were subsequently transfected into Weri-RB1 cells. 2.5 × 10^6^ transfected Weri-RB1 cells were resuspended in 100 μL PBS and injected into the axillae of the mice. Tumor size was measured with calipers as (length × width^2^)/2. After 20 days, mice were euthanized with carbon dioxide, tumors were removed and weighed.

### Statistical analysis

All detection experiments are repeated 3 times, and the measurement data is expressed as mean ± SD. SPSS 21.0 software was used for statistical analysis. The two groups were compared by *t*-test, and the comparisons between three and more different groups were performed by the one-way analysis of variance LSD method. The difference was statistically significant when P < 0.05.

## Results

### Knockdown of Bmi-1 restrains cell proliferation, migration and invasion, and increases cell apoptosis

**Figure 1:**
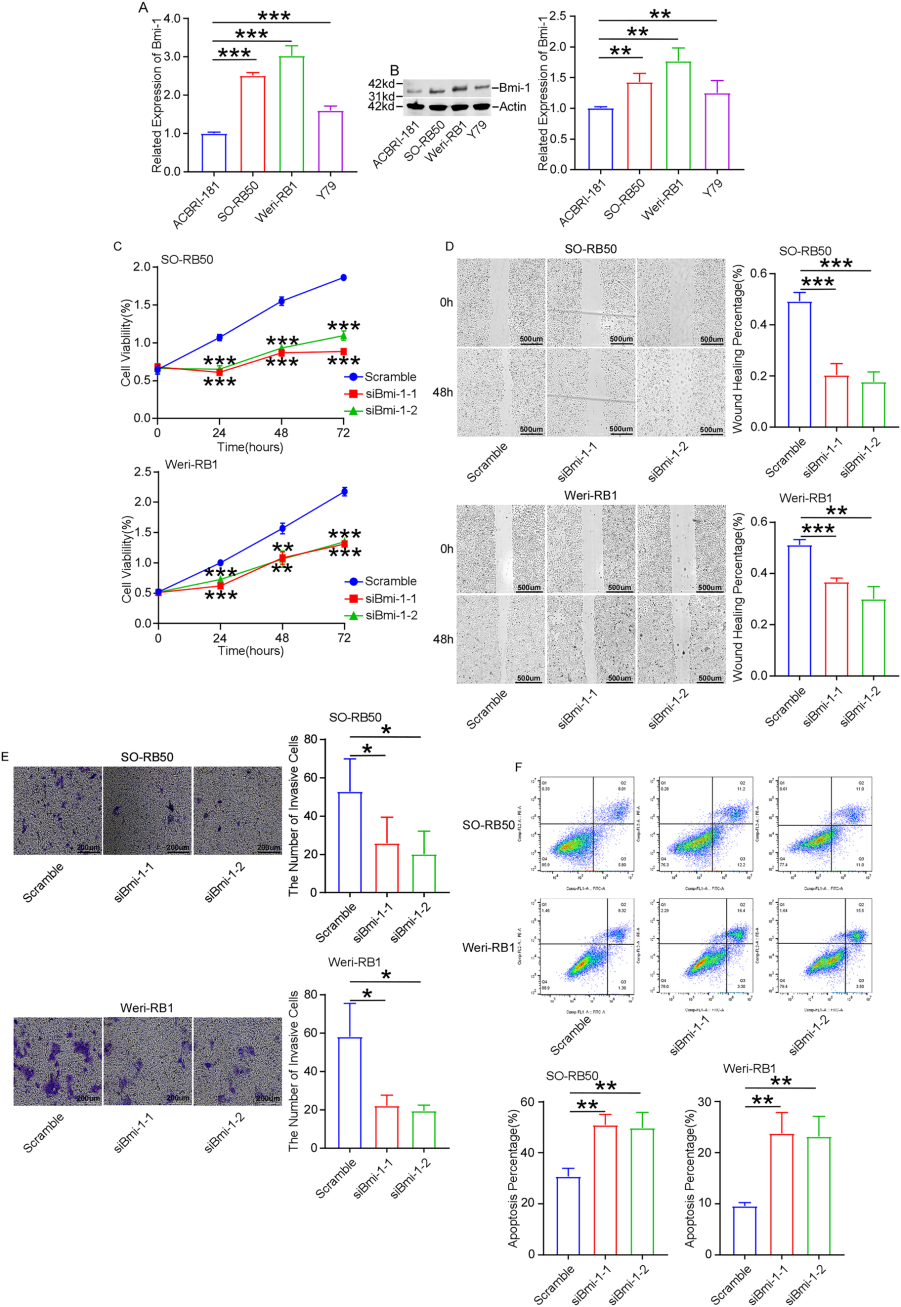
Knockdown of Bmi-1 inhibits cell proliferation, migration and invasion, and increases cell apoptosis. RT-qPCR (**A**) and Western blot (**B**) showed that the expression of Bmi-1 was higher in Y79, SO-RB50 and Weri-RB1 cells than that of normal retinal vascular endothelial cell line ACBRI-181. (**C**) MTT assay revealed that knockdown of Bmi-1 suppressed cell proliferation compared with control group at 24 h, 48 h and 72 h. (**D**) Wound healing displayed that Bmi-1 silencing reduced the migration of SO-RB50 cells (Scale bar: 500 μm) and Weri-RB1 cells (Scale bar: 500 μm). (**E**) Transwell assay displayed that Bmi-1 silencing reduced the invasion of SO-RB50 cells and Weri-RB1 cells (Scale bar: 200 μm). (**F**) FCM demonstrated that knockdown of Bmi-1 increased cell apoptosis. *P < 0.05, **P < 0.01, ***P < 0.001, compared to the ACBRI-181 cells or the Scramble group.

To explore the roles of Bmi-1, the expression of Bmi-1 in retinoblastoma cell lines using RT-qPCR and Western blot. As expected, the expression of Bmi-1 was overexpressed in retinoblastoma cell lines Y79, SO-RB50 and Weri-RB1 than that of normal retinal vascular endothelial cell line ACBRI-181 (Fig. 1A,B).

To investigate the effect of Bmi-1 on tumor progression, siRNA-Bmi-1 was used to knockdown Bmi-1 in SO-RB50 and Weri-RB1 cells. The knockout efficiency was measured by RT-qPCR and Western blot (Supplementary Fig. 1A,B). MTT assay were used to measure the cell proliferation. MTT assay revealed that knockdown of Bmi-1 suppressed cell proliferation compared with control group at 24 h, 48 h and 72 h (Fig. 1C). Wound healing and Transwell assays were applied to detect the migration and invasion abilities. The results demonstrated that cell migration and invasion was reduced when silence of Bmi-1 in comparison with control (Fig. 1D,E). Cell apoptosis was measured using FCM. The results demonstrated that knockdown of Bmi-1 increased cell apoptosis (Fig. 1F). These findings revealed that inhibition of Bmi-1 suppressed cell proliferation, migration and invasion, and increased cell apoptosis in retinoblastoma.

### Overexpression of Bmi-1 promotes cell proliferation, migration and invasion, and inhibits cell apoptosis

**Figure 2:**
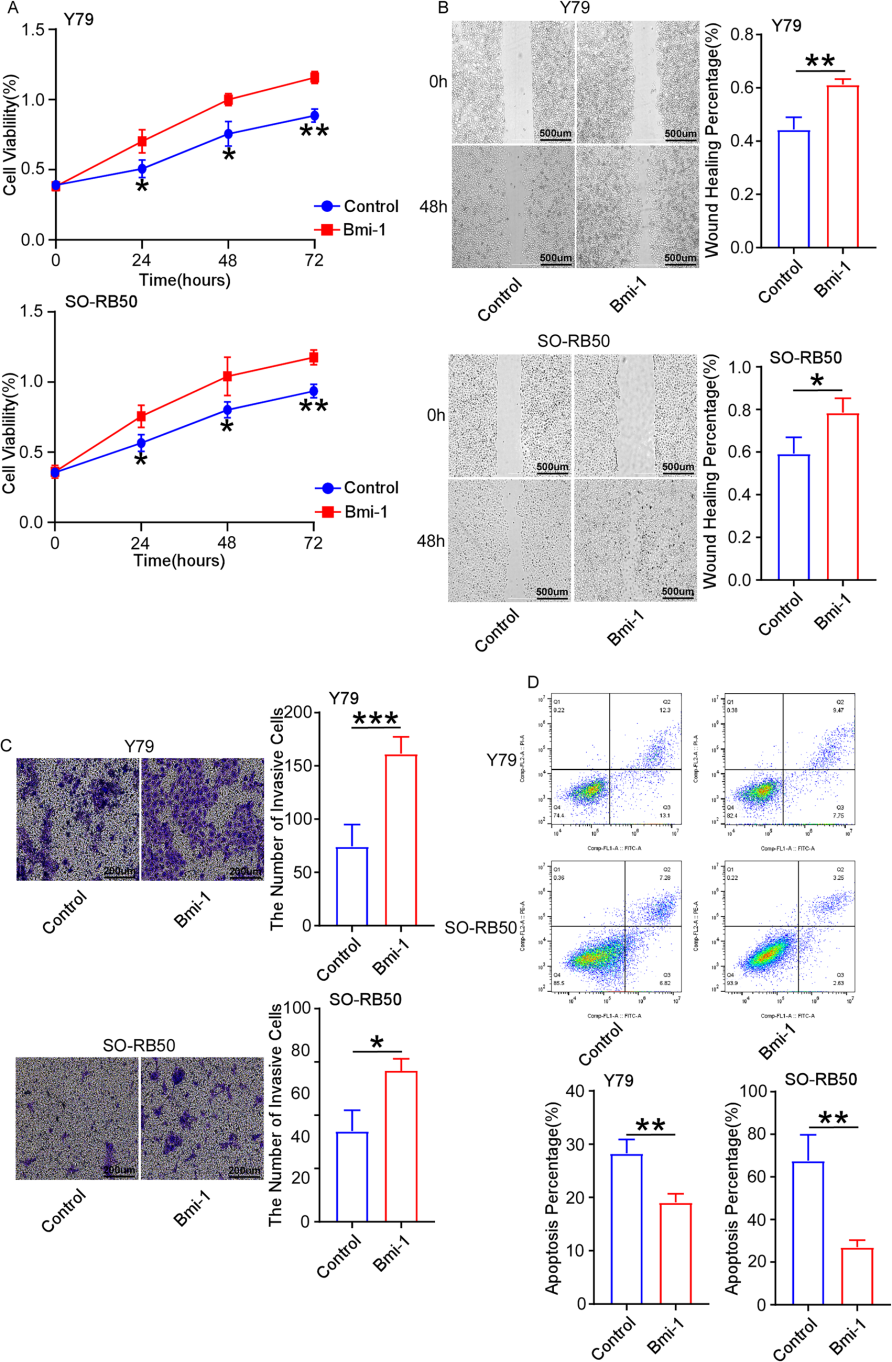
Overexpression of Bmi-1 promotes cell proliferation, migration and invasion, and restrains cell apoptosis. (**A**) MTT assay indicated that cell proliferation was improved by overexpressing Bmi-1 at 24 h, 48 h and 72 h. (**B**,**C**) Overexpression of Bmi-1 increased cell migratory (scale bar: 500 μm) and invasive capacities (scale bar: 200 μm). (**D**) FCM results illustrated that overexpression of Bmi-1 inhibited cell apoptosis. *P < 0.05, **P < 0.01, ***P < 0.001, compared to the Control group.

**Figure 3:**
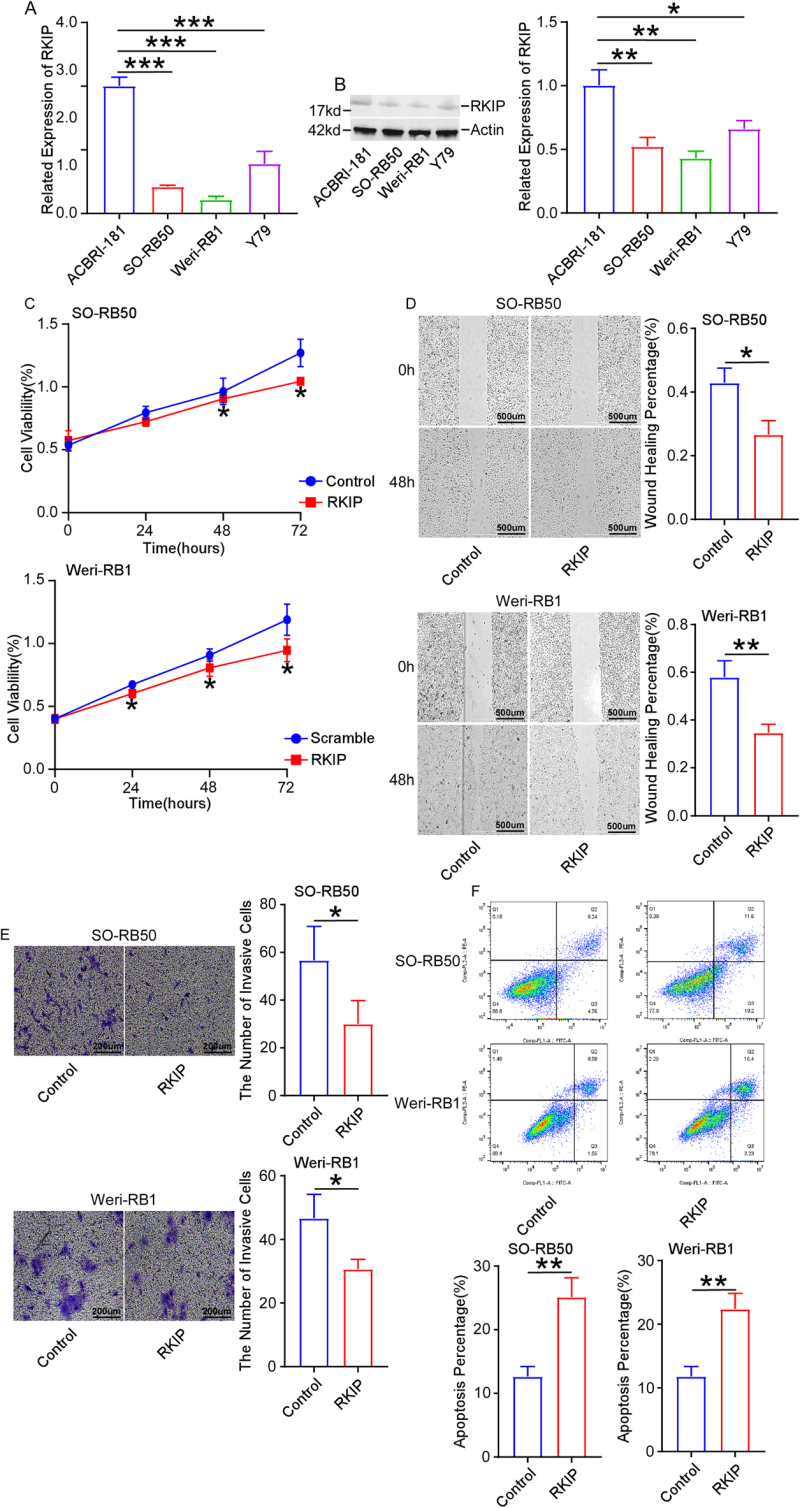
Overexpression of RKIP suppresses cell proliferation, migration and invasion, increases cell apoptosis. (**A**,**B**) The expression of RKIP was lower in retinoblastoma cells Y79, SO-RB50 and Weri-RB1 than that of normal retinal vascular endothelial cell ACBRI-181. (**C**–**E**) Cell proliferation, migration (scale bar: 500 μm) and invasion (scale bar: 200 μm) were inhibited after overexpressing RKIP in cells. (**F**) Cell apoptosis were promoted after overexpressing RKIP in cells. *P < 0.05, **P < 0.01, ***P < 0.001, compared to the ACBRI-181 cells or the Control group.

Bmi-1 was upregulated by transfecting overexpressed plasmid in Y79 and SO-RB50 cells, and the transfection efficiency was also detected by RT-qPCR and Western blot (Supplementary Fig. 2A,B). MTT assay indicated that cell proliferation was improved by overexpressing Bmi-1 at 26 h, 48 h and 72 h (Fig. 2A). Wound healing and Transwell assays revealed that overexpression of Bmi-1 increased cell migratory and invasive capacities (Fig. 2B,C). Overexpression of Bmi-1 inhibited cell apoptosis (Fig. 3D). These findings revealed that Bmi-1 increased cell proliferation, migration and invasion, and suppressed cell apoptosis in retinoblastoma.

### Overexpression of RKIP suppresses cell proliferation, migration and invasion, increases cell apoptosis

Previous studies demonstrated that RKIP was a downstream gene of Bmi-1^18^. The expression of RKIP was calculated in retinoblastoma cells using RT-qPCR and western blot. As expected, the expression of RKIP was lower in Y79, SO-RB50 and Weri-RB1 cells than that of normal retinal vascular endothelial cell ACBRI-181 (Fig. 3A,B). To explore the roles of RKIP in retinoblastoma, the expression of RKIP was conducted to upregulate in SO-RB50 and Weri-RB1 cells, and the transfection efficiency was measured using RT-qPCR and western blot (Supplementary Fig. 3A,B). As expected, cell proliferation, migration and invasion were inhibited after overexpressing RKIP in SO-RB50 and Weri-RB1 cells (Fig. 3C–E). Overexpression RKIP increased cell apoptosis (Fig. 3F). All the findings revealed that overexpression of RKIP suppressed cell proliferation, migration and invasion, and increased cell apoptosis in retinoblastoma.

### RKIP knockdown suppresses the functions of Bmi-1 knockdown on retinoblastoma cells

**Figure 4:**
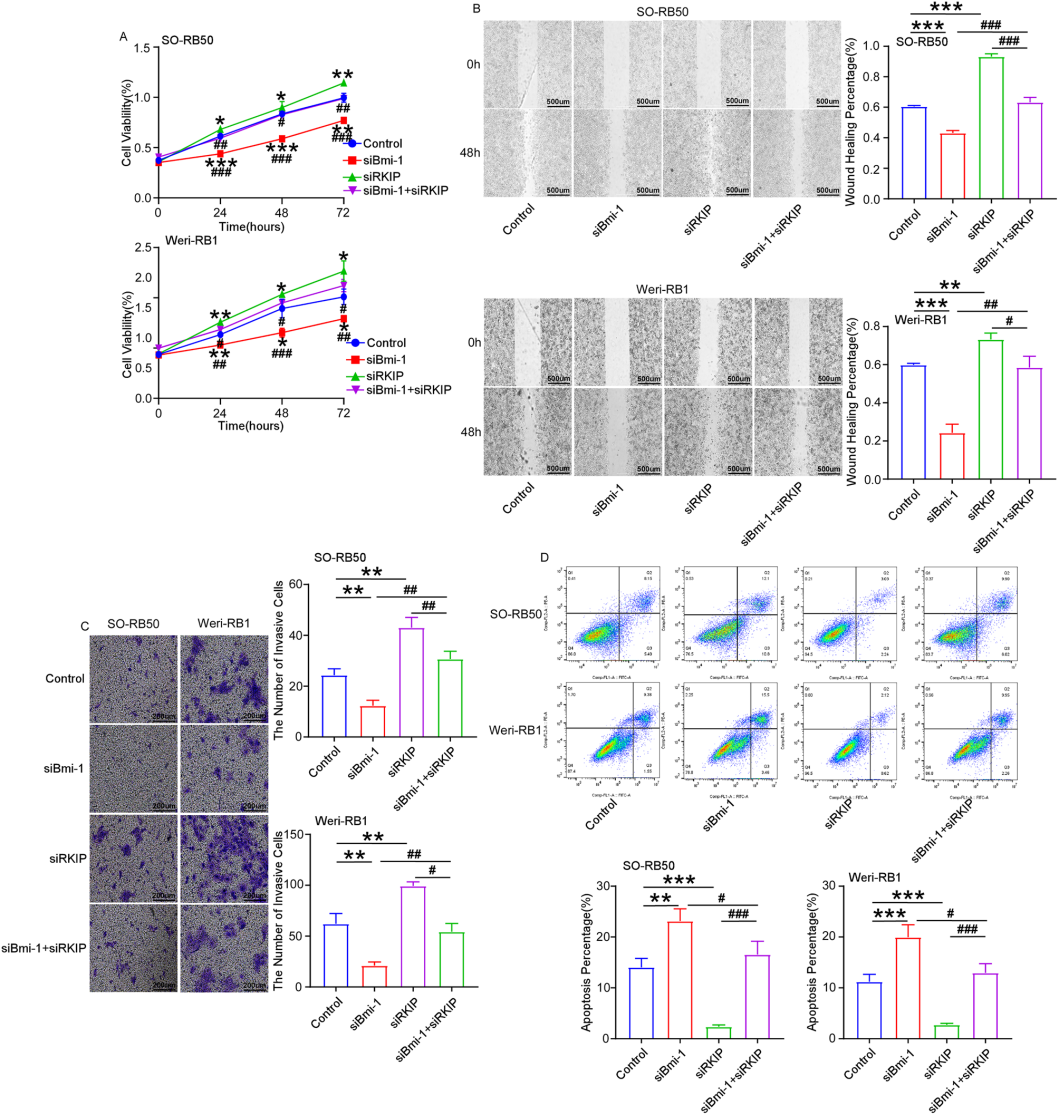
RKIP knockdown suppresses the functions of Bmi-1 knockdown on retinoblastoma cells. (**A**) MTT assay revealed that RKIP knockdown accelerated cell proliferation and reversed the inhibitory effect of siRNA-Bmi-1 on cell proliferation at 24 h, 48 h, and 72 h. (**B**,**C**) Wound healing and Transwell assay revealed that RKIP knockdown reversed the inhibitory effect of siRNA-Bmi-1 on cell migration (scale bar: 500 μm) and invasion (scale bar: 200 μm). (**D**) FCM assay showed that RKIP knockdown suppressed the pro-apoptotic effect of siRNA-Bmi-1. *P < 0.05, **P < 0.01, ***P < 0.001, compared to the Control group; ^#^*P* < 0.05, ^##^*P* < 0.01, ^###^*P* < 0.001, compared to the siBmi-1+siRKIP group.

To investigate the effect of RKIP on tumor progression, siRNA- RKIP was used to knockdown RKIP in SO-RB50 and Weri-RB1 cells. The knockout efficiency was measured by RT-qPCR and Western blot (Supplementary Fig. 4A,B). siRNA-RKIP accelerated cell proliferation and reversed the inhibitory effect of siRNA-Bmi-1 on cell proliferation at 24 h, 48 h, and 72 h (Fig. 4A). Moreover, siRNA-RKIP promoted cell migration and invasion, and reversed the inhibition of siRNA-Bmi-1 on cell migration and invasion (Fig. 4B,C). Furthermore, siRNA-RKIP suppressed cell apoptosis and inhibited the pro-apoptotic effect of siRNA-Bmi-1 (Fig. 4D). The findings revealed that RKIP knockdown suppressed the role of Bmi-1 knockdown in retinoblastoma cells.

### RKIP increases the functions of Bmi-1 knockdown on retinoblastoma cells

**Figure 5:**
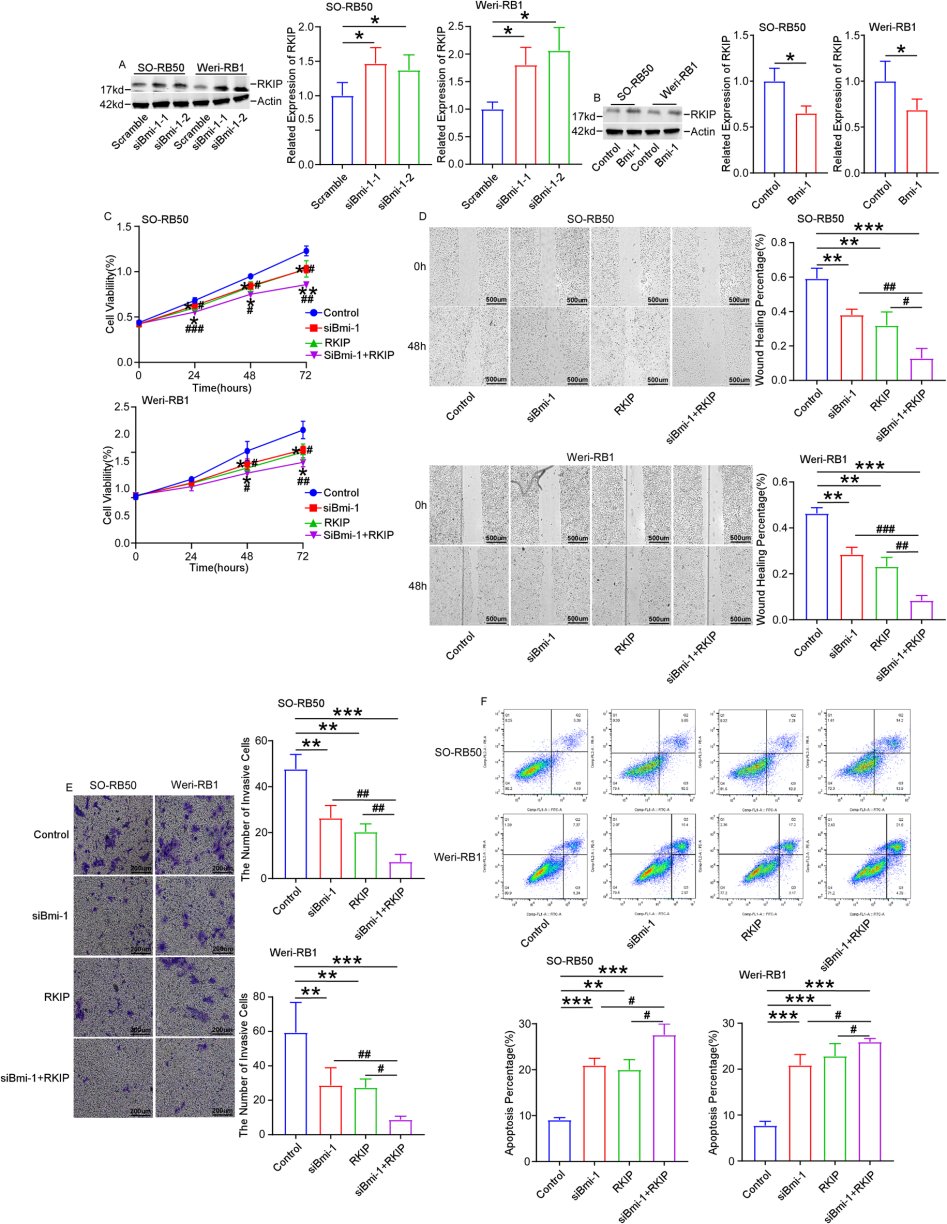
RKIP increases the functions of Bmi-1 knockdown on retinoblastoma cells. (**A**) The expression of RKIP was upregulated after knockdown of Bmi-1. (**B**) RKIP was downregulated after overexpressing Bmi-1 in cells. (**C**) Cell proliferation of cells co-transfected with RKIP and siRNA-Bmi-1 at 24 h, 48 h and 72 h were more decreased compared with cells only transfected siRNA-Bmi-1. (**D**,**E**) Cell migration (scale bar: 500 μm) and invasion (scale bar: 200 μm) of cells co-transfected with RKIP and siRNA-Bmi-1 were more diminished relative to cells transfected siRNA-Bmi-1. (**F**) Cell apoptosis of cells co-transfected with RKIP and siRNA-Bmi-1 were more increased relative to cells transfected siRNA-Bmi-1. *P < 0.05, **P < 0.01, ***P < 0.001, compared to the Scramble group or the Control group; ^#^*P* < 0.05, ^##^*P* < 0.01, ^###^*P* < 0.001, compared to the siBmi-1+RKIP group.

In addition, the expression of RKIP was upregulated after Bmi-1 knockdown in SO-RB50 and Weri-RB1 cells (Fig. 5A). On the contrary, RKIP was downregulated after overexpressing Bmi-1 (Fig. 5B). The cell proliferation of SO-RB50 and Weri-RB1 cells co-transfected with RKIP and siRNA-Bmi-1 at 24 h, 48 h and 72 h were decreased compared with cells only transfected siRNA-Bmi-1 or RKIP (Fig. 5C). Similarly, the migration and invasion ability of cells co-transfected with RKIP and siRNA-Bmi-1were lowest in all groups (Fig. 5D,E). The apoptosis of cells co-transfected with RKIP and siRNA-Bmi-1 were highest in all groups (Fig. 5F). All the findings revealed the functions of Bmi-1/RKIP axis in retinoblastoma tumorigenesis.

### Downregulation of Bmi-1 suppresses xenograft tumor growth by regulating RKIP in vivo

**Figure 6:**
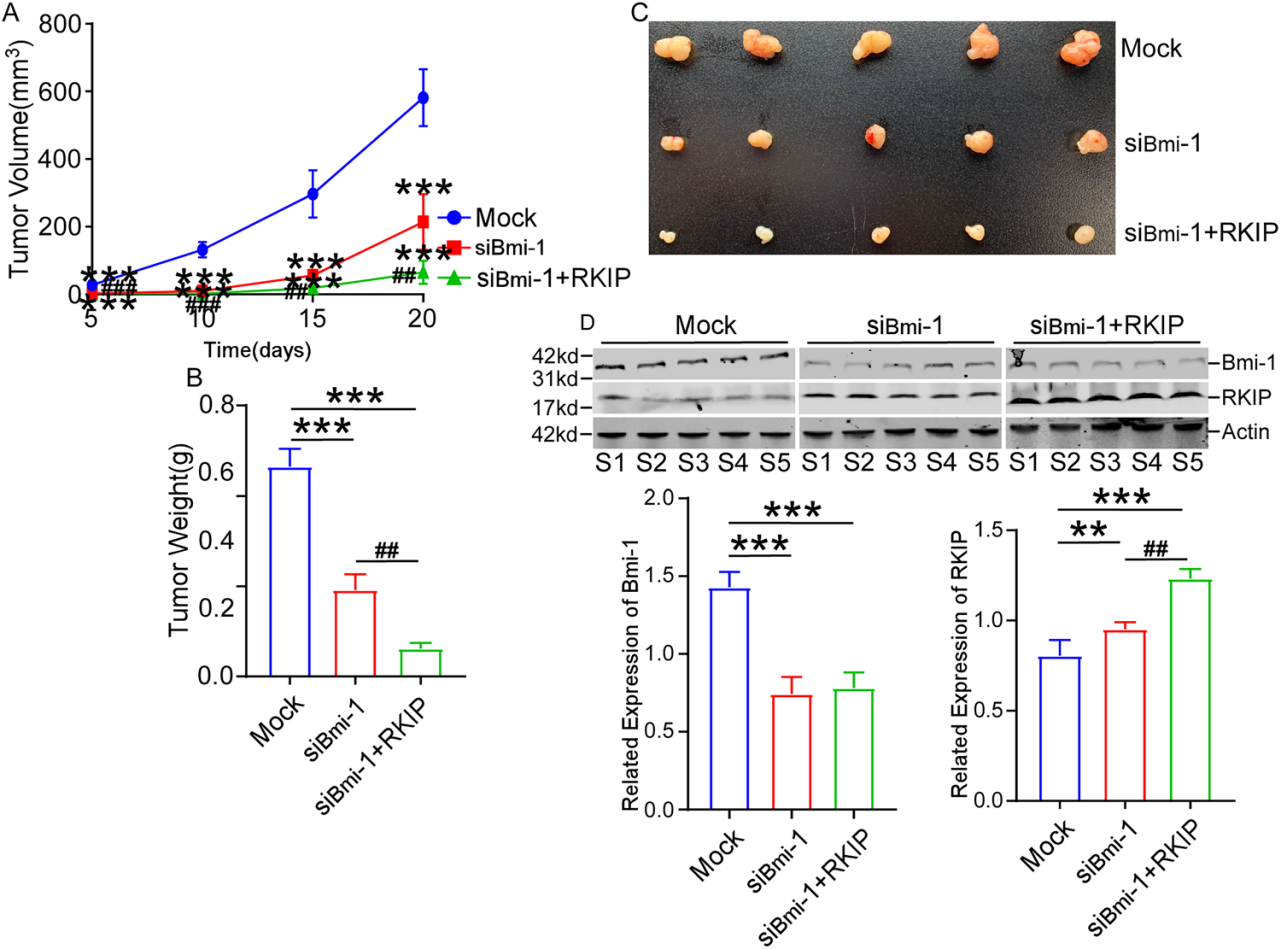
Downregulation of Bmi-1 suppresses xenograft tumor growth by regulating RKIP in vivo. (**A**) Tumor growth was significantly attenuated after subcutaneous injection of siRNA-Bmi-1, and the volume of transplanted tumor of mice injected with siRNA-Bmi-1 and RKIP were more downregulated relative to those only injected with siRNA-Bmi-1. (**B**,**C**) The volume and weight of transplanted tumors were significantly suppressed in mice injected with siRNA-Bmi-1 compared to controls. Moreover, the volume and weight of transplanted tumors were more smaller in mice injected with siRNA-Bmi-1 and RKIP than in mice injected with siRNA-Bmi-1 only. (**D**) Bmi-1 expression was low, and RKIP expression was high in tumor tissues transfected with siRNA-Bmi-1 and in tumor tissues co-transfected with siRNA-Bmi-1 and RKIP. *P < 0.05, **P < 0.01, ***P < 0.001, compared to the Mock group; ^##^*P* < 0.01, ^###^*P* < 0.001, c﻿ompared ﻿to the siBmi-1+RKIP group.

To detect whether Bmi-1 and RKIP affect retinoblastoma tumorigenesis in vivo, nude mice were injected with Weri-RB1 cells transfected with siRNA-Bmi-1 alone or co-transfected siRNA-Bmi-1 with RKIP. The volume of the subcutaneous transplanted tumor was measured every 5 days, starting on day 5 after injection. Tumor growth was significantly attenuated after subcutaneous injection of siRNA-Bmi-1. What’s more, the volume of transplanted tumor of mice injected with siRNA-Bmi-1 and RKIP were more downregulated relative to those only injected with siRNA-Bmi-1 (Fig. 6A). At day 20, mice were euthanized and the weighed and volume of tumors were calculated. The volume and weight of transplanted tumors were significantly suppressed in mice injected with siRNA-Bmi-1 compared to controls. Moreover, the volume and weight of transplanted tumors were smaller in mice injected with siRNA-Bmi-1 and RKIP than in mice injected with siRNA-Bmi-1 only (Fig. 6B,C). Bmi-1 expression was low in tumor tissues transfected with siRNA-Bmi-1 and in tumor tissues co-transfected with siRNA-Bmi-1 and RKIP (Fig. 6D). RKIP expression was high in tumor tissues transfected with siRNA-Bmi-1 and in tumor tissues co-transfected with siRNA-Bmi-1 and RKIP (Fig. 6D). These results suggested that downregulation of Bmi-1 inhibited tumor growth in vivo by regulating RKIP.

## Discussion

Retinoblastoma is a common ocular malignant tumor in infants and young children. The global incidence of retinoblastoma is about 1/15,000, with about 9000 new cases per year^19^. The mortality rate of retinoblastoma was 40–70% in Asia and Africa, and 3–5% in developed countries in Europe and United States^20^. Retinoblastoma seriously endangers the eyesight and life of children, and brings great pain to children and their families. Therefore, it is of great significance to improve the diagnosis and treatment of retinoblastoma.

Bmi-1 induces the malignant transformation, which has been reported to be associated with tumor size, clinical stage and prognosis of gastric cancer^14,21^. Similarly, the modulation of BMI-1 leads to DNA damage, M phase cell cycle arrest, chromosome scattering and cell death in glioma^22^. What’s more, BMI-1 expression is increased in early oral carcinogenesis and is possibly associated with the occurrence of dysplastic changes^23^. Consistent with the above findings, we found that Bmi-1 was overexpressed in retinoblastoma tissues and retinoblastoma cells. In addition, knockdown of Bmi-1 suppressed cell viability, invasion and migration, and increased cell apoptosis. Moreover, tumor transplantation experiments in nude mice showed that knocking down Bmi-1 inhibited tumor growth in vivo.

Raf-1 kinase inhibitory protein (RKIP) is a prototypical member of the phosphatidylethanolamine-binding protein family, and is initially identified as a Raf1^24,25^. RKIP emerges as a well-known suppressor in carcinogenesis^26^. Increasing evidences have shown that RKIP is downregulated in several cancers, including breast cancer, gastric cancer, colorectal cancer and prostate cancer^27–30^. Knockdown of RKIP enhances nasopharyngeal carcinoma invasion and metastasis by activating Stat3 signaling^31^. In breast cancer and prostate cancer, RKIP blocks signal transducer and activator of transcription 3 activation^32^. In this study, we found that RKIP was low expressed in retinoblastoma tissues and retinoblastoma cells. Moreover, overexpression of RKIP inhibited retinoblastoma cell progression in vitro and tumor growth in vivo. What’s more, overexpression of RKIP partially enhanced the roles of knockdown of Bmi-1. Hence, all results suggested that downregulation of Bmi-1 suppressed cell progression by regulating RKIP.

## Conclusion

Bmi-1 promoted retinoblastoma cell progression through regulating the expression of RKIP. Bmi-1 serves as a promising therapy target for improving the efficacy of clinical treatment in retinoblastoma.

## Supplementary Information

Supplementary Information.

Supplementary Figure 1.

Supplementary Figure 2.

Supplementary Figure 3.

Supplementary Figure 4.

Supplementary Legends.
