# Author Correction: Bmi-1 promotes the proliferation, migration and invasion, and inhibits cell apoptosis of human retinoblastoma cells via RKIP

**DOI:** 10.1038/s41598-025-11879-x

**Published:** 2025-08-05

**Authors:** Qian Li, Te Fu, Ning Wei, Qiaoling Wang, Xin Zhang

**Affiliations:** Department of Ophthalmology, The Second People’s Hospital of Jinan, No. 148, Jingyi Road, Jinan, 250000 Shandong China

Correction to: *Scientific Reports* 10.1038/s41598-024-65011-6, published online 24 June 2024

The original version of the Article contained an error in Figure 1. As a result of an error during figure assembly, the image for the Scramble condition for SO-RB50 in Figure 1E was mistakenly a duplication of the image for the siBmi-1 + siRKIP for SO-RB50 in Figure 4C.

The original Figure 1 and accompanying legend appear below.

**Fig. 1:**
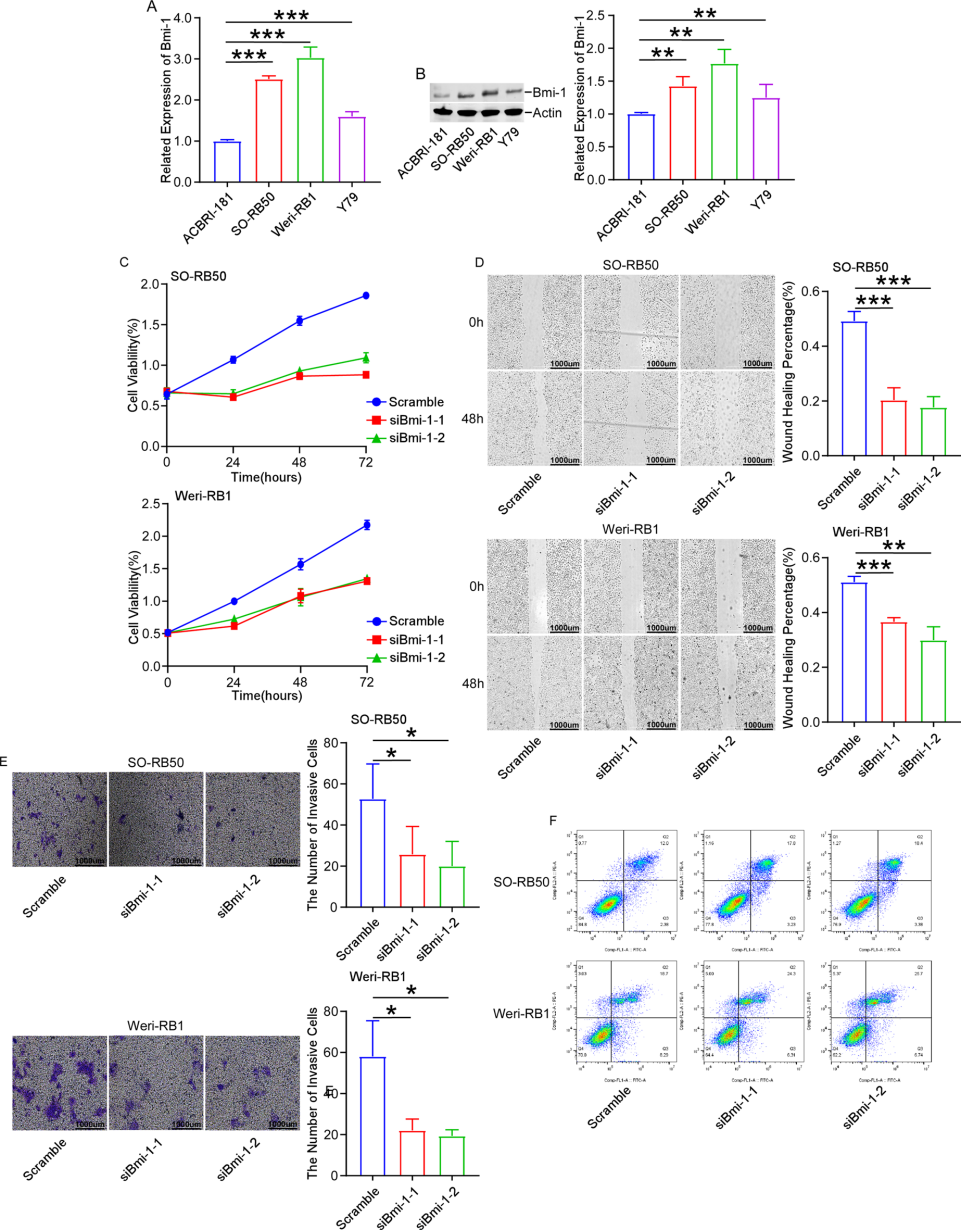
Knockdown of Bmi-1 inhibits cell proliferation, migration and invasion, and increases cell apoptosis. RT-qPCR (**A**) and Western blot (**B**) showed that the expression of Bmi-1 was higher in Y79, SO-RB50 and Weri-RB1 cells than that of normal retinal vascular endothelial cell line ACBRI-181. (**C**) MTT assay revealed that knockdown of Bmi-1 suppressed cell proliferation compared with control group at 24 h, 48 h and 72 h. (**D**) Wound healing displayed that Bmi-1 silencing reduced the migration of SO-RB50 cells (Scale bar: 500 μm) and Weri-RB1 cells (Scale bar: 500 μm). (**E**) Transwell assay displayed that Bmi-1 silencing reduced the invasion of SO-RB50 cells and Weri-RB1 cells (Scale bar: 200 μm). (**F**) FCM demonstrated that knockdown of Bmi-1 increased cell apoptosis. *P < 0.05, **P < 0.01, ***P < 0.001, compared to the ACBRI-181 cells or the Scramble group.

The original Article has been corrected.

