# Supplementary material for: Bmi-1 promotes the proliferation, migration and invasion, and inhibits cell apoptosis of human retinoblastoma cells via RKIP
Source: Sci Rep. 2024 Jun 24;14:14544. doi: 10.1038/s41598-024-65011-6 (PMC11196667; doi:10.1038/s41598-024-65011-6)
Supplement: Supplementary file 1 — Supplementary Information. [file 41598_2024_65011_MOESM1_ESM.docx]

**Fig1B Bmi-1**

**
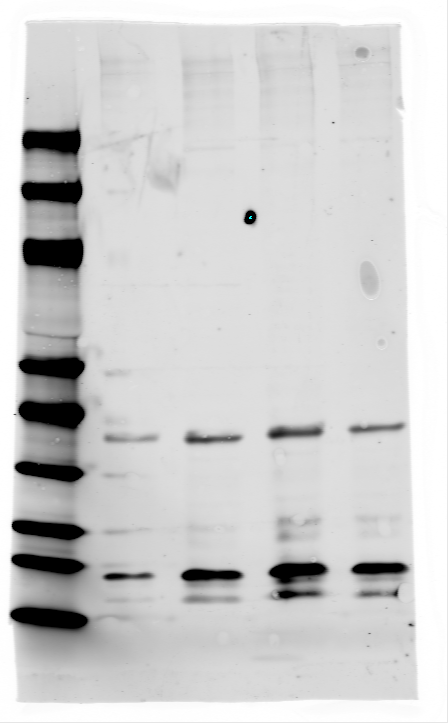
**

**Fig1B actin**

**
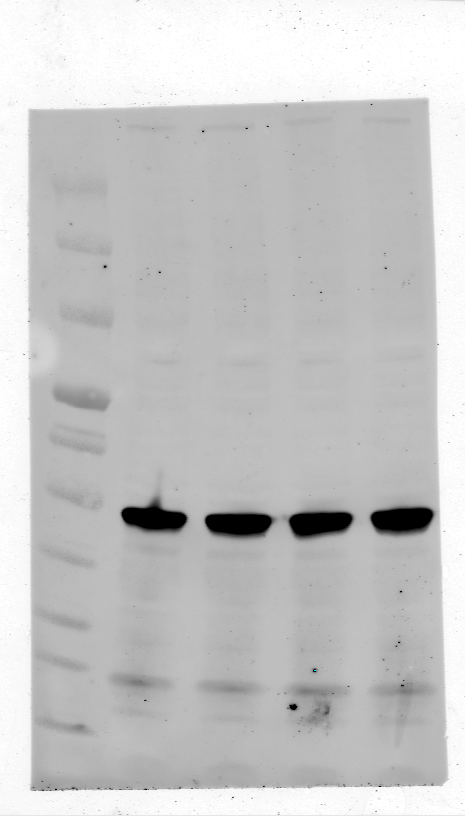
**

**Fig3B RKIP**

**

**

**Fig3B actin**

**
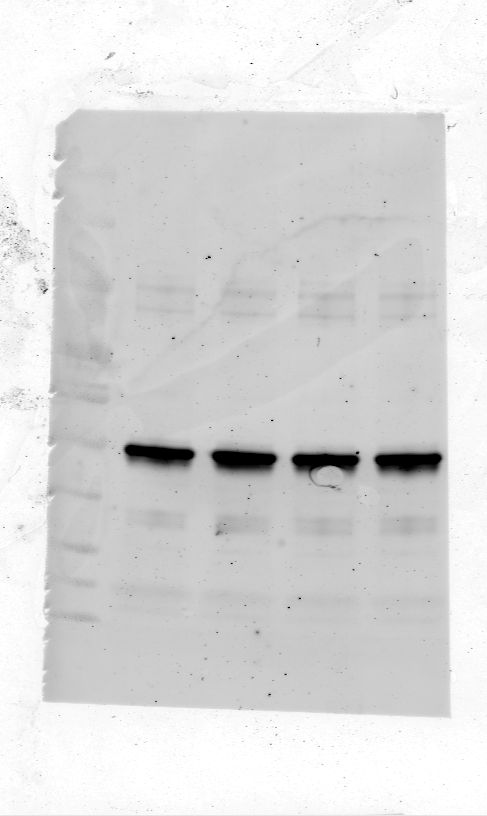
**

**Fig5A RKIP**

**
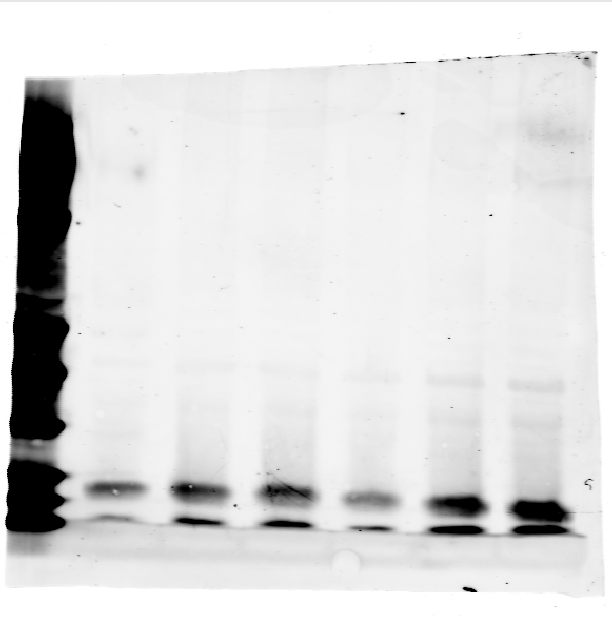
**

**Fig5A actin**

**
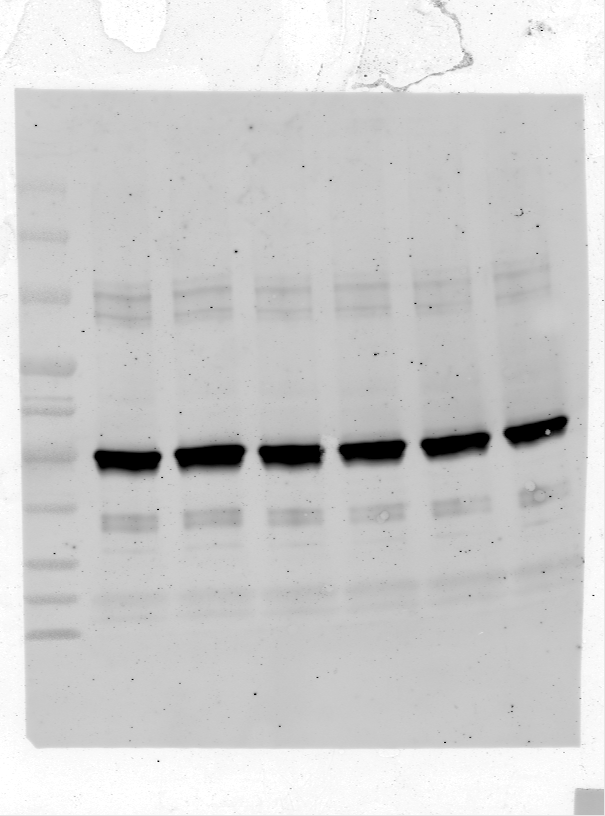
**

**Fig5B RKIP**

**
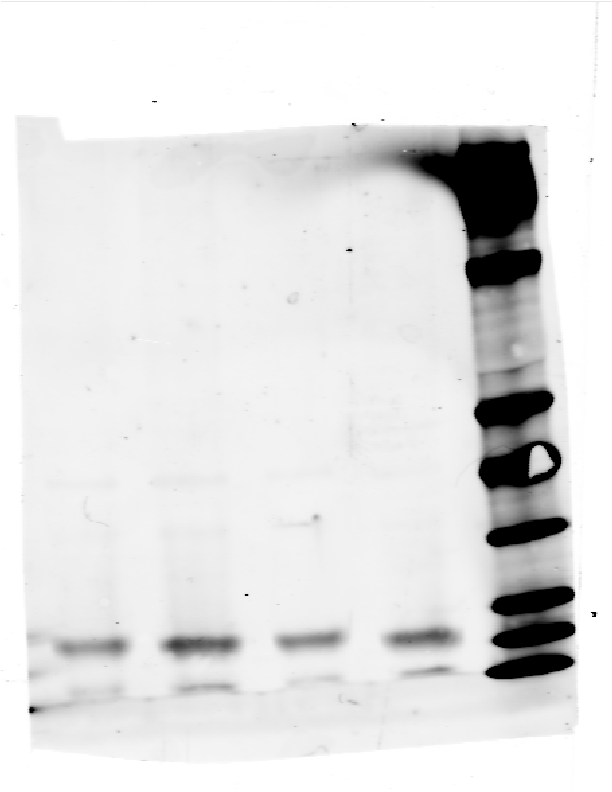
**

**Fig5B actin**

**
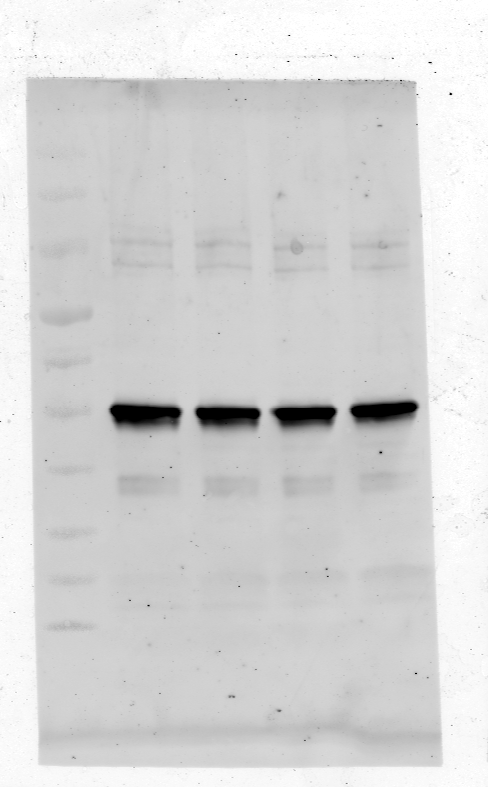
**

**Fig6D BMI-1**

**

**

**Fig6D RKIP**

**
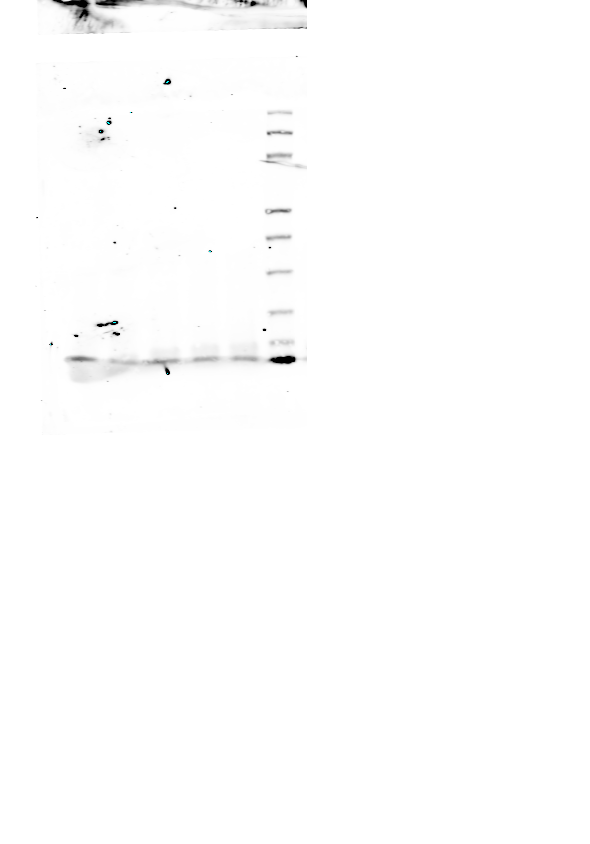
**

**

 Fig6D actin**

**

**

**Sup. Fig1 Bmi-1**

**
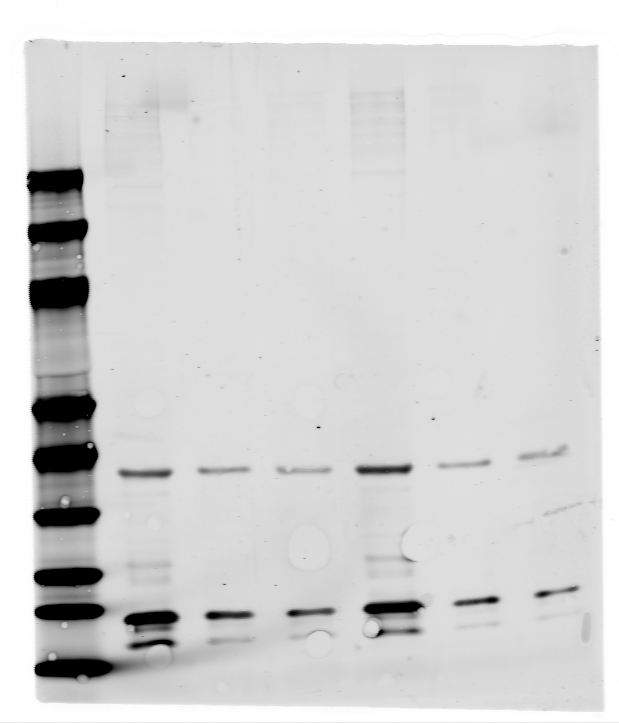
**

**Sup. Fig1 actin**

**
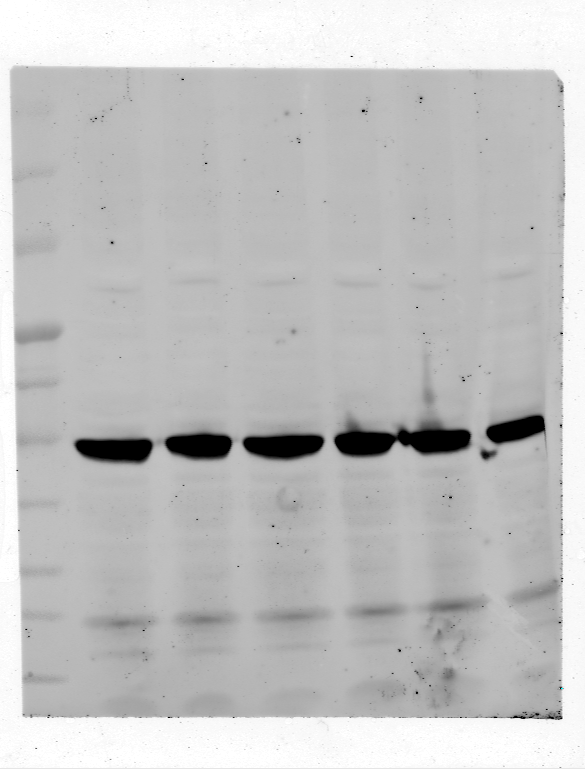
**

**Sup. Fig2 Bmi-1**

**
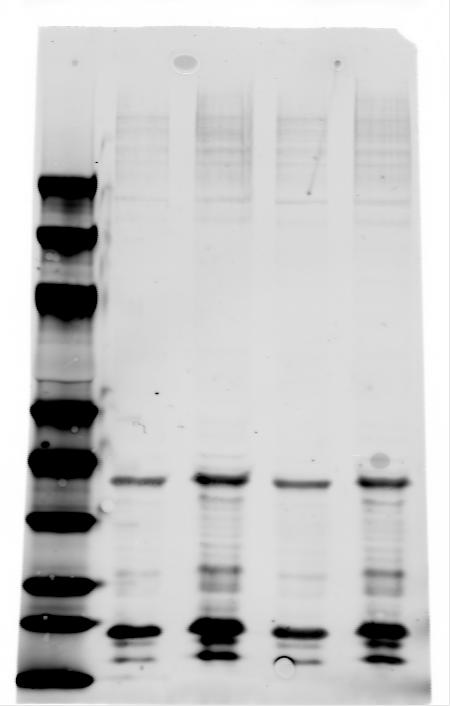
**

**Sup. Fig2 actin**

**
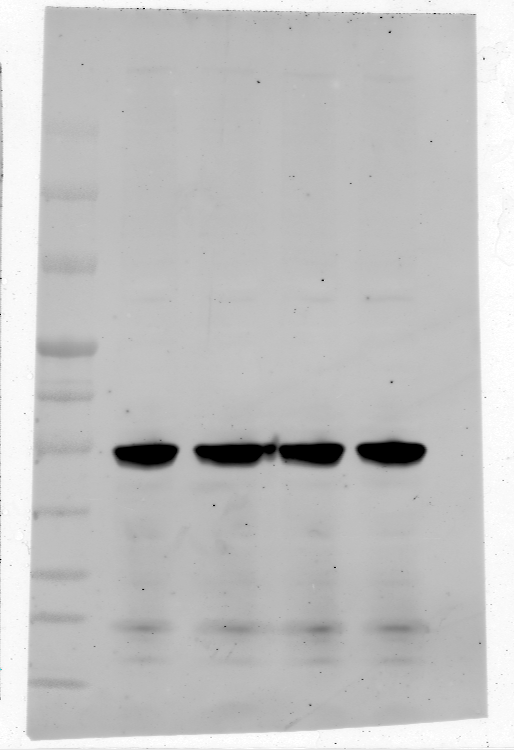
**

**Sup. Fig3 RKIP**

**
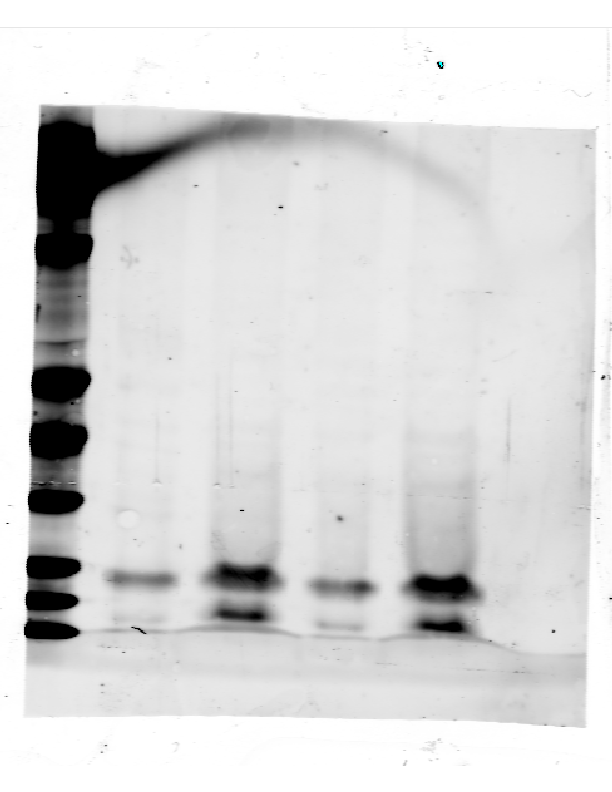
**

**Sup. Fig3 actin**

**
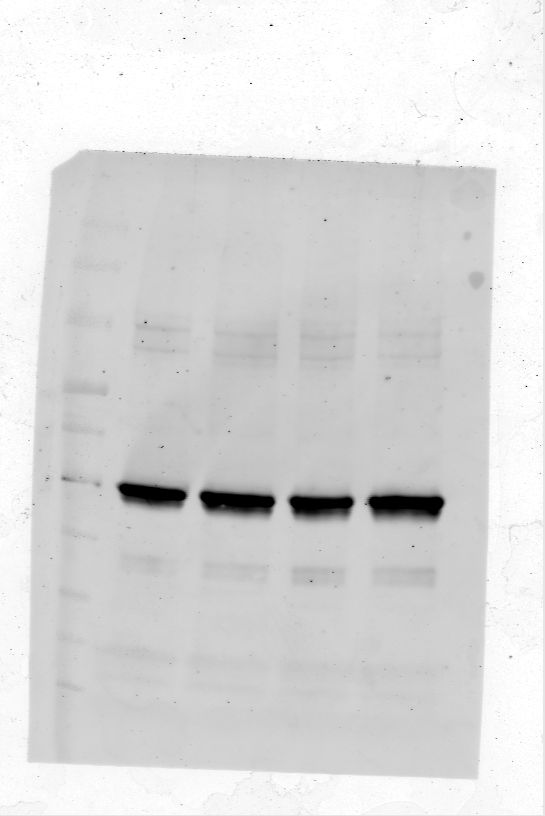
**

**Sup. Fig4 RKIP**

**

**

**Sup. Fig4 actin**

**
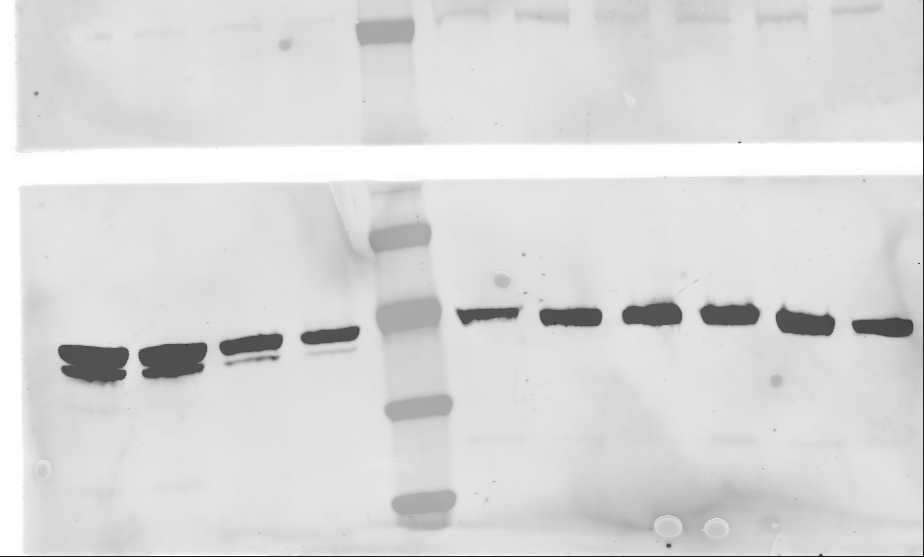
**
