# Supplementary figures and images for: Bmi-1 promotes the proliferation, migration and invasion, and inhibits cell apoptosis of human retinoblastoma cells via RKIP
Source: Sci Rep. 2024 Jun 24;14:14544. doi: 10.1038/s41598-024-65011-6 (PMC11196667; doi:10.1038/s41598-024-65011-6)

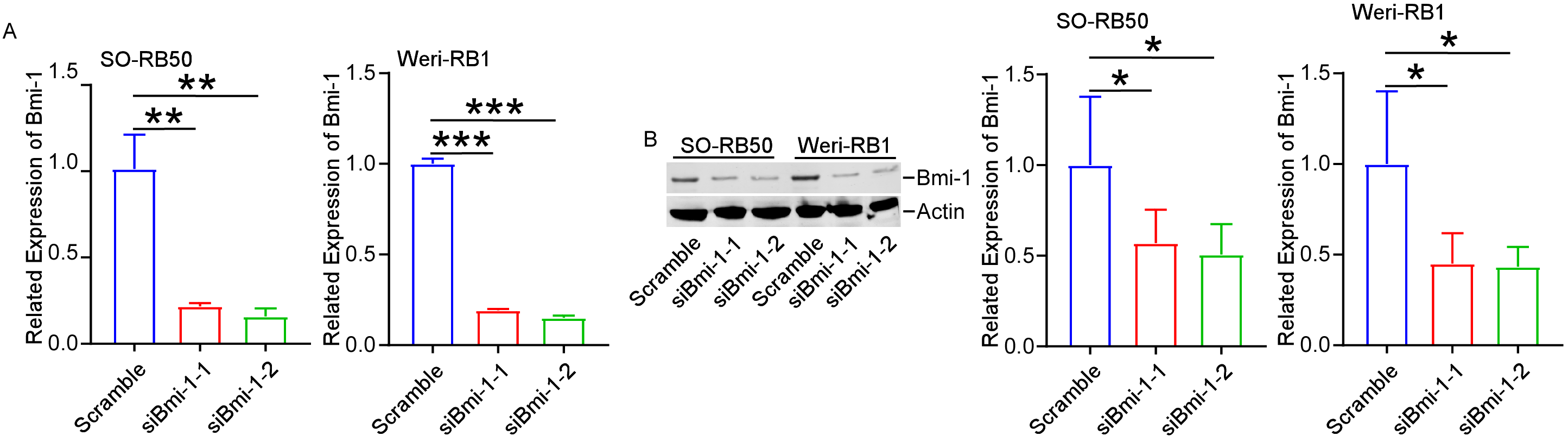

Supplement: Supplementary file 2 — Supplementary Figure 1. [file 41598_2024_65011_MOESM2_ESM.tif]

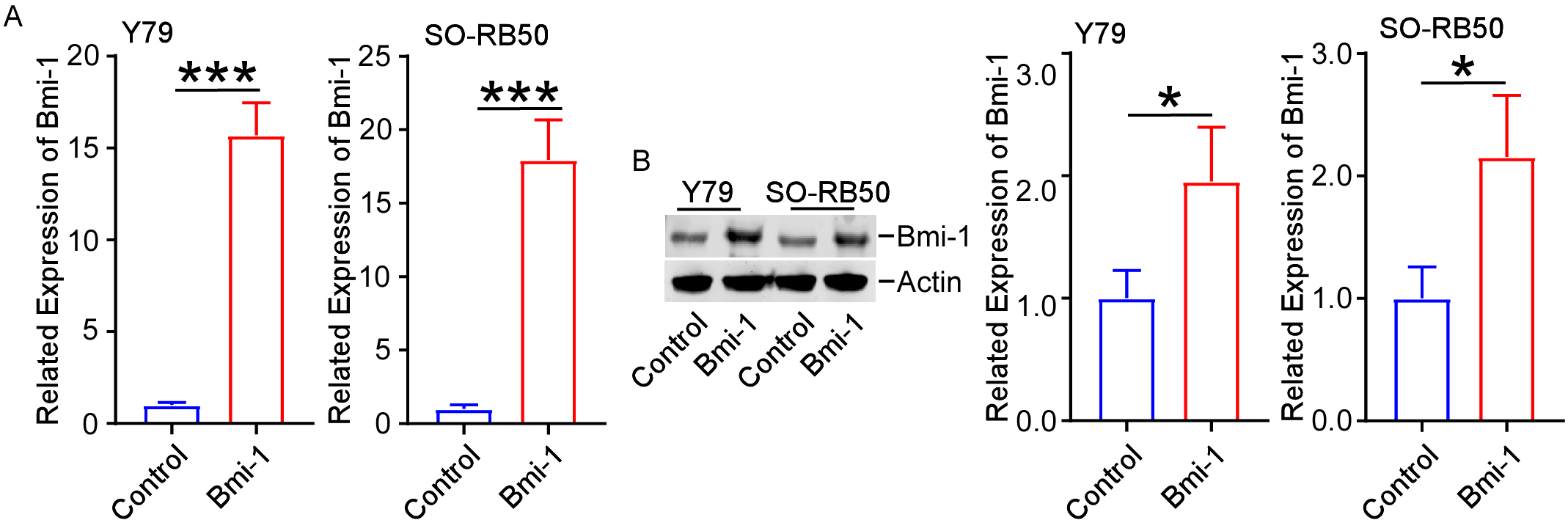

Supplement: Supplementary file 3 — Supplementary Figure 2. [file 41598_2024_65011_MOESM3_ESM.tif]

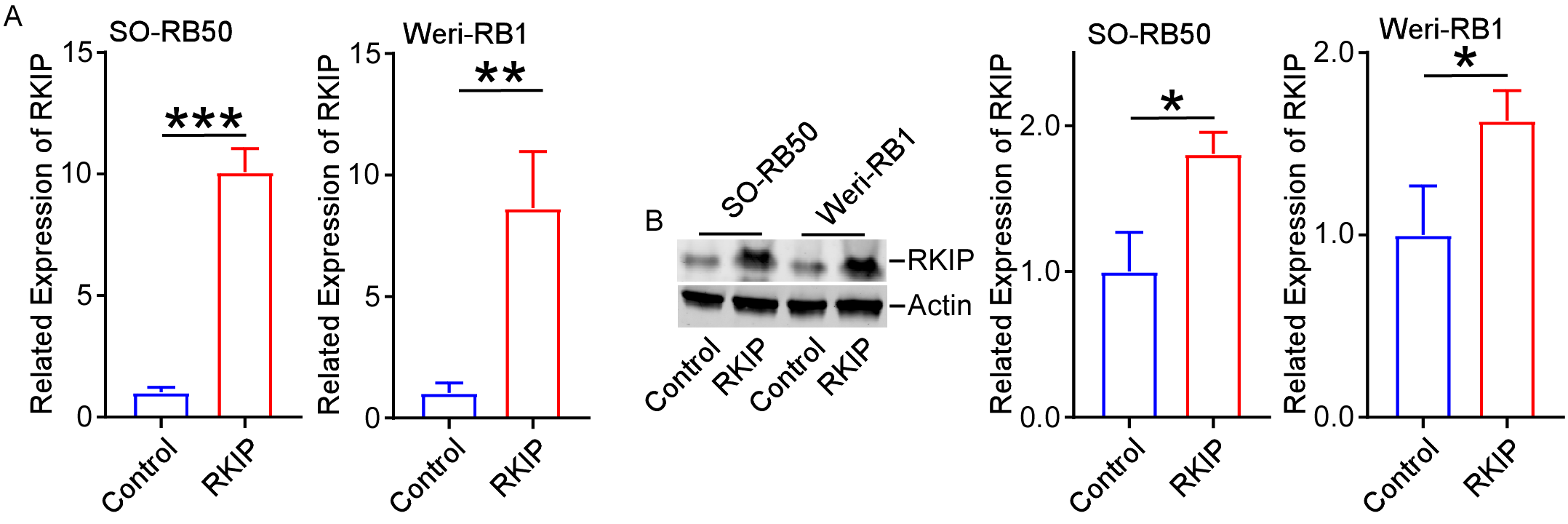

Supplement: Supplementary file 4 — Supplementary Figure 3. [file 41598_2024_65011_MOESM4_ESM.tif]

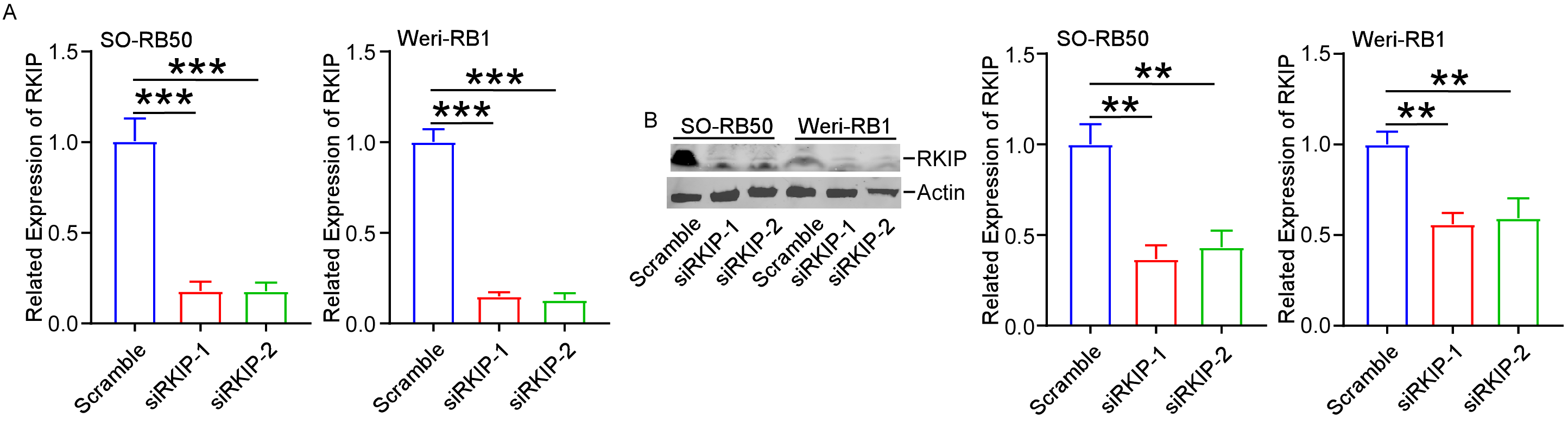

Supplement: Supplementary file 5 — Supplementary Figure 4. [file 41598_2024_65011_MOESM5_ESM.tif]
