# Supplementary material for: Bmi-1 promotes the proliferation, migration and invasion, and inhibits cell apoptosis of human retinoblastoma cells via RKIP
Source: Sci Rep. 2024 Jun 24;14:14544. doi: 10.1038/s41598-024-65011-6 (PMC11196667; doi:10.1038/s41598-024-65011-6)
Supplement: Supplementary file 6 — Supplementary Legends. [file 41598_2024_65011_MOESM6_ESM.docx]

**Supplementary Figure 1** The knockout efficiency of Bmi-1 knockdown in cells.

(A, B). The efficiency was measured by RT-qPCR and Western blot. *P < 0.05, **P < 0.01, ***P < 0.001, compared to the Scramble group.

**Supplementary Figure 2** The transfection efficiency of upregulating Bmi-1 in cells.

(A, B). The efficiency was measured by RT-qPCR and Western blot. *P < 0.05, **P < 0.01, ***P < 0.001, compared to the Control group.

**Supplementary Figure 3** The transfection efficiency of upregulating RKIP in cells.

(A, B). The efficiency was measured by RT-qPCR and Western blot. *P < 0.05, **P < 0.01, ***P < 0.001, compared to the Control group.

**Supplementary Figure 4** The knockout efficiency of RKIP knockdown in cells.

(A, B) The efficiency was measured by RT-qPCR and Western blot. *P < 0.05, **P < 0.01, ***P < 0.001, compared to the Scramble group.
